# PNImodeler: web server for inferring protein-binding nucleotides from sequence data

**DOI:** 10.1186/1471-2164-16-S3-S6

**Published:** 2015-01-29

**Authors:** Jinyong Im, Narankhuu Tuvshinjargal, Byungkyu Park, Wook Lee, De-Shuang Huang, Kyungsook Han

**Affiliations:** 1 Department of Computer Science and Engineering, Inha University, Incheon, South Korea; 2 Machine Learning and Systems Biology Lab, College of Electronics and Information Engineering, Tongji University, Shanghai 201804, China

## Abstract

**Background:**

Interactions between DNA and proteins are essential to many biological processes such as transcriptional regulation and DNA replication. With the increased availability of structures of protein-DNA complexes, several computational studies have been conducted to predict DNA binding sites in proteins. However, little attempt has been made to predict protein binding sites in DNA.

**Results:**

From an extensive analysis of protein-DNA complexes, we identified powerful features of DNA and protein sequences which can be used in predicting protein binding sites in DNA sequences. We developed two support vector machine (SVM) models that predict protein binding nucleotides from DNA and/or protein sequences. One SVM model that used DNA sequence data alone achieved a sensitivity of 73.4%, a specificity of 64.8%, an accuracy of 68.9% and a correlation coefficient of 0.382 with a test dataset that was not used in training. Another SVM model that used both DNA and protein sequences achieved a sensitivity of 67.6%, a specificity of 74.3%, an accuracy of 71.4% and a correlation coefficient of 0.418.

**Conclusions:**

Predicting binding sites in double-stranded DNAs is a more difficult task than predicting binding sites in single-stranded molecules. Our study showed that protein binding sites in double-stranded DNA molecules can be predicted with a comparable accuracy as those in single-stranded molecules. Our study also demonstrated that using both DNA and protein sequences resulted in a better prediction performance than using DNA sequence data alone. The SVM models and datasets constructed in this study are available at http://bclab.inha.ac.kr/pnimodeler.

## Background

Interactions between nucleic acids and proteins have diverse functions within a cell, and play an important role in many biological processes. For example, proteins that bind to specific regions of DNA act as transcription factors by activating or repressing gene expression of the DNA. Thus, identifying protein recognition parts in DNAs or DNA recognition parts in proteins will help understand a variety of cellular processes [1,2]. As many structures of protein-DNA complexes have been determined, theoretical and experimental studies have been conducted in recent years to study protein-DNA interactions, but protein-DNA interactions and their mechanisms are not yet fully understood.

Several computational methods have been developed to predict DNA- or RNA-binding residues in protein sequences using machine learning methods such as support vector machines (SVM) as classifiers. BindN [1] uses SVM to predict RNA- or DNA-binding residues in proteins based on the biochemical features of amino acids. DP-Bind [3] predicts DNA-binding residues in proteins and uses SVM with a position specific scoring matrix (PSSM) and amino acid properties. DNABindR [4] uses a naïve Bayes classifier to predict DNA-binding residues in proteins. MetaDBSite [5] predicts DNA-binding residues by integrating the prediction results from six predictors (DISIS, DNABindR, BindN, BindN-rf, DP-Bind and DBS-PRED). A method developed by Liu *et al*. [6] predicts RNA-binding sites in proteins using a random forest. It uses several features such as mutual interaction propensity, physicochemical characteristics, hydrophobicity, relative accessible surface area, secondary structure, conservation score and side-chain environment.

Instead of finding DNA-binding sites in proteins, some works have attempted to classify whether a given protein is DNA-binding or non-binding. iDNA-Prot [7] classifies proteins into DNA-binding and non-binding proteins from amino acid sequence data. DBPPred [8] also classifies whether a given protein is a DNA-binding protein or not using secondary structure, relative solvent accessibility and PSSM.

Several studies have been conducted to find effective features of proteins in predicting DNA-binding sites in proteins. For example, Yi *et al*. [9] characterized DNA-binding residues on protein surface with B-factors and packing density, and Dey *et al*. [10] investigated evolutionary conservation, spatial clustering, hydrogen bond donor capability and residue propensity.

Unlike the previous works that have focused on DNA- or RNA- binding proteins, in the present work we attempted to predict protein binding nucleotides using sequence data. Predicting protein binding sites in DNA is more difficult than predicting DNA binding sites in proteins for several reasons: (1) for a sequence of the same length, DNA has many fewer sequence patterns than protein, (2) in protein-DNA interactions nucleotides show less diverse interaction propensities than amino acids, and (3) predicting binding sites in a double-stranded molecule (i.e., DNA) is more complicated than predicting binding sites in a single protein sequence.

In the present work we studied key features of DNA and protein sequences and their representation to predict protein binding sites in DNA. We developed two SVM models and compared their performances with actual data. One SVM model (hereafter called DPI1) predicts binding sites in a given DNA sequence with unknown protein. Another SVM model (called DPI2) predicts binding sites in a given DNA sequence with a specified protein. Experimental results showed that the SVM model that used DNA sequence data alone predicted more binding sites than the SVM model that used both DNA and protein sequences, but the overall performance of the latter was higher than that of the former. In this paper, we present our approach to the problem of predicting protein binding nucleotides from sequence data and discuss experimental results.

## Methods

### Definition of a binding site

**Figure 1:**
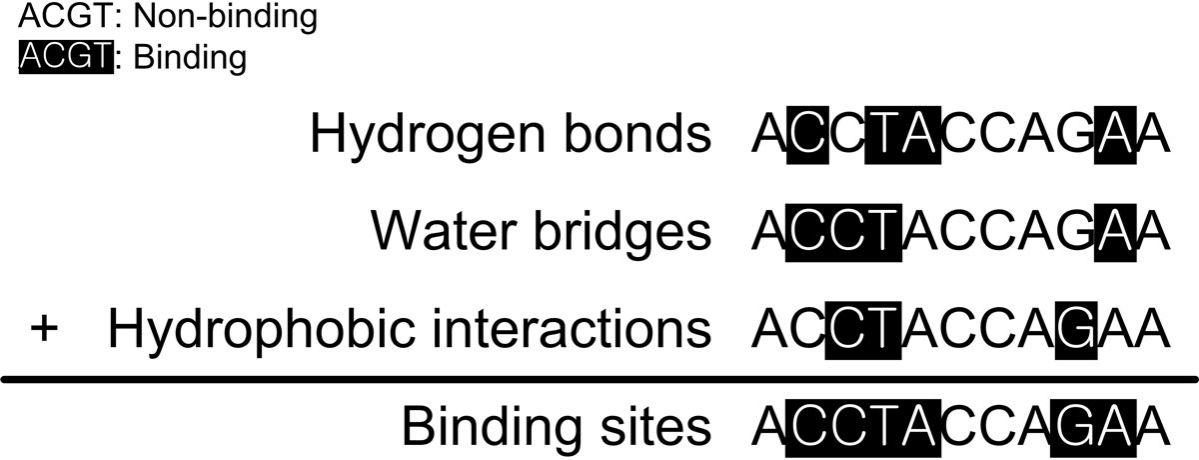
**Criteria of protein binding nucleotides**. If a nucleotide is involved in any of the protein-DNA interactions of 3 types (hydrogen bonds, water bridges and hydrophobic interactions), it is classified as a binding site. A nucleotide in a black background represents a protein-binding site and others represent non-binding sites.

Several types of interactions are involved in protein-DNA interactions, and different studies use different criteria to define a binding site in protein-DNA interactions [1,3,4]. In this study of protein-DNA interactions, we considered three types of interactions to define a binding site: hydrogen bonds, water bridges and hydrophobic interactions. We obtained protein-DNA pairs involved in the three types of interactions from the nucleic acid-protein interaction database (NPIDB) [11]. A nucleotide participating in any of the interactions of three types were classified as a binding site as shown in Figure 1.

### Dataset

We collected protein-DNA complexes which are determined by X-ray crystallography with a resolution of 3.0 Å or better from the Protein Data Bank (PDB) [12]. As of July 2013, there were 1,654 protein-DNA complexes which contain 1,589 DNA sequences and 892 protein sequences.

We divided the 1,589 DNA sequences into two groups using CD-HIT-EST [13]. 1,416 DNA sequences with the similarity of 80% or higher were selected as a training dataset for the prediction models. The remaining 173 DNA sequences that have a similarity lower than 80% with any sequence of the training dataset were used as a test dataset for the prediction models. We applied the feature vector-based method to the 1,416 DNA sequences to construct a non-redundant training dataset. The feature vector-based redundancy removal method, developed in our previous study [14], constructs a larger training dataset of non-redundant data than the standard sequence similarity-based reduction method. The initial 1,416 DNA sequences of the training dataset form 2,658 interaction pairs with 837 protein sequences, and the 173 DNA sequences of the test dataset form 189 interaction pairs with 135 protein sequences.

Our prediction models do not assume that the structure data or sequence direction is known, so they handle each sequence in double stranded DNA molecules separately to predict protein-binding sites in the DNA sequence. The training dataset for the model that uses both DNA and protein sequences contains 20,588 binding nucleotides and 27,630 non-binding nucleotides. For the model that uses only DNA sequence data, binding sites in a same DNA sequence with different protein partners were incorporated in the DNA sequence. Thus, the training dataset for this model contains fewer binding and non-binding nucleotides (20,378 binding nucleotides and 23,950 non-binding nucleotides) than that for the model that uses both DNA and protein sequences.

### Support Vector Machines and Feature Vectors

We implemented two prediction models using a library for support vector machines (LIBSVM) [15]. As a kernel function we used the radial basis function (RBF). Two parameters (cost and gamma) of RBF control the performance and time-cost. We found the best values of the parameters cost and gamma for each window size using an optimization tool called grid.py. We assigned binding nucleotides a weight of 1.3 to balance the data size of binding nucleotides with that of non-binding nucleotides.

Since SVM handles numerical data, we encode sequence information into a feature vector with numerical elements. We created feature values from three types of sequence data: original DNA sequence, DNA sequence fragments from the original DNA sequence, and protein sequence interacting with the DNA. The original DNA sequence is represented by its base composition. DNA sequence fragments are represented by nucleotide triplet composition, normalized position, molecular mass, molecular pK_a _and interaction propensity (IP) of nucleotide triplets [14]. For protein, which is an interaction partner of DNA, we represent the sum of normalized position of 20 amino acids [14] and dipeptide composition [16].

The base composition represents the percentage of four nucleotides in a DNA sequence (equation 1). The nucleotide triplet com-position represents the frequency of a nucleotide triplet using a sequence encoding scheme called the n-gram extraction method [17]. For a given sequence, the n-gram method extracts the patterns and frequencies of n consecutive nucleotides. There are 64 (= 4 × 4 × 4) possible nucleotide triplets, and they are represented by 64 features in a feature vector using equation 2. The IP represents the binding propensities between nucleotide triplets and amino acids. The normalized position of the *i*-th nucleotide or amino acid is its relative position in the original sequence (equation 3).

The dipeptide composition is the frequency of 400 (= 20 × 20) amino acid duplet patterns [16]. The partner feature represents the sum of the normalized positions of 20 amino acids (equation 4).

**Figure 2:**
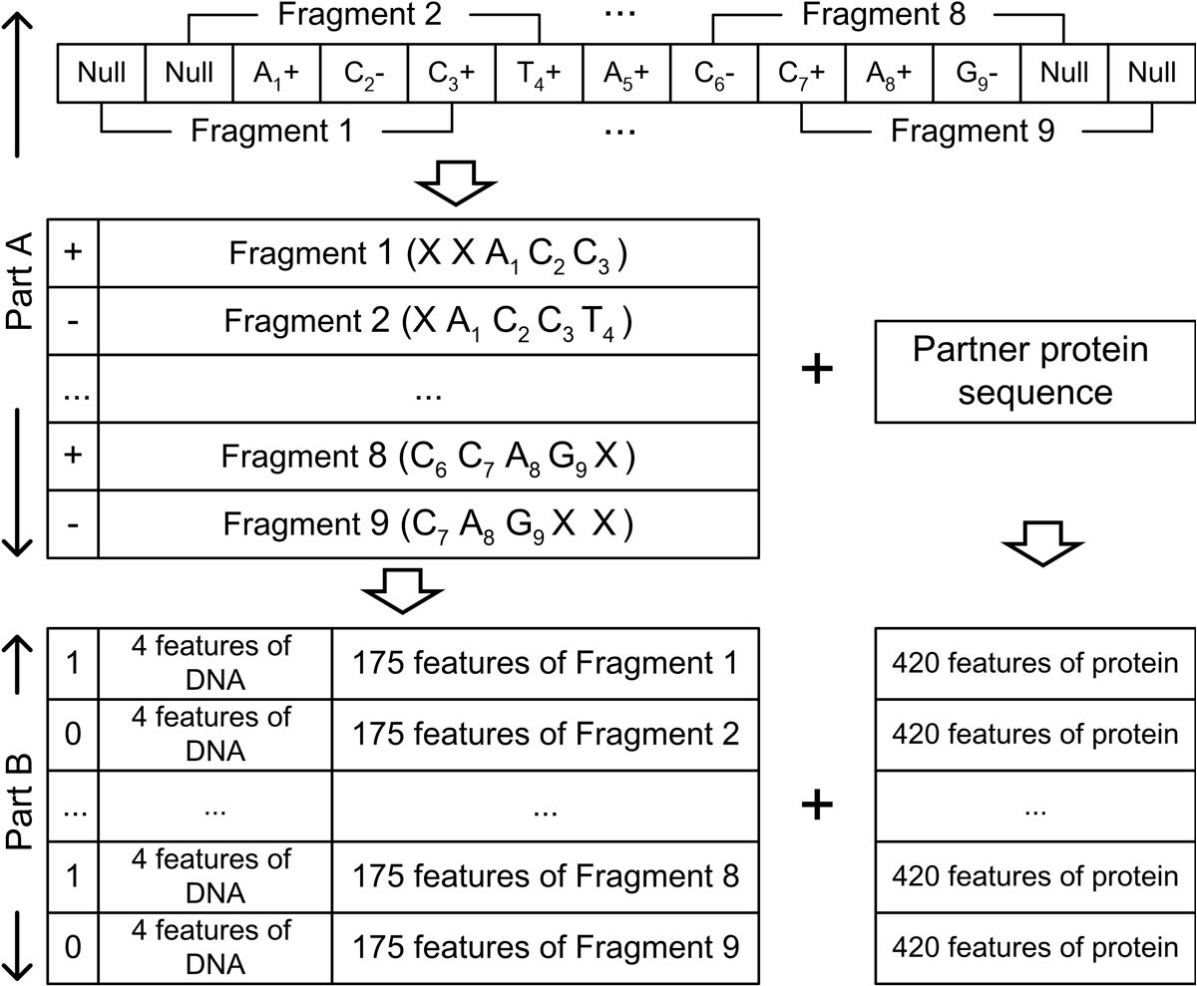
**Example of representing a DNA sequence of 9 nucleotides by a sliding window of size 5**. The '+' symbol represents binding, and the '-' symbol represents non-binding. 'X' in fragments indicates a null nucleotide at the position. Part A explains dividing a DNA sequence into sequence fragments and Part B explains encoding a sequence fragment in a feature vector. For each DNA sequence fragment of 5 nucleotides, 179 elements are encoded in a feature vector: 4 elements for the base composition, 5 elements for mass, 5 elements for pK**_a_**, 1 element for the normalized position, 64 elements for the nucleotide triplet composition, and 100 elements for the IP of nucleotide triplets. The features of protein are encoded in 420 feature elements (20 elements for the sum of the normalized positions of 20 amino acids + 400 elements for the dipeptide composition). The feature vector for DPI1 does not include the 420 features of protein.

A feature vector representing a sequence fragment of length S consists of 4 elements for the base composition, S elements for mass, S elements for pK_a_, 1 element for the normalized position, 64 elements for the triplet composition and 20×S elements for the IP of nucleotide triplets. For example, a DNA sequence fragment of 5 nucleotides is encoded by 179 feature elements (4 elements for the base composition + 5 elements for mass + 5 elements for pK_a _+ 1 element for the normalized position + 64 elements for the nucleotide triplet composition + 100 elements for the IP of nucleotide triplets). In addition, 420 feature elements (20 elements for the sum of the normalized positions of 20 amino acids + 400 elements for the dipeptide composition) are included in a feature vector to represent a partner protein sequence for DPI2 as shown in Part B of Figure 2.

(1) $$\mathrm{Base}\;\mathrm{composition}{\left(b\right)}_{b\in \left\{A,C,G,T\right\}}=\frac{\mathrm{Total}\;\mathrm{occurrences}(\text{b})}{\mathrm{Sequence}\;\mathrm{length}}$$

(2) $$\text{Triplet}\;\text{composition}{\left(t\right)}_{\text{t}\in \left\{64\;\text{triplets}\right\}}=\frac{\text{Total}\;\text{occurences}\left(t\right)}{64}$$

(3) $$\text{Normalized}\;\text{position}\left(i\right)=\frac{\text{Position}\left(i\right)}{\text{Sequence}\;\text{length}}$$

(4) $$\text{Partner}\;\text{feature}{\left(a\right)}_{\text{a}\in \left\{20\;\;\text{amino}\;\text{acids}\right\}}=\overset{\text{Length}}{\underset{i,a_{i}=a}{\sum }}\text{Normalized}\;\text{position(}a\text{)}$$

A DNA sequence is represented by overlapping sequence fragments using a sliding window method. Part A of Figure 2 shows the process of dividing sequences with a DNA sequence of length 9 and sliding window of size 5. After we represented the sequence fragments, we removed redundant feature vectors using the feature vector-based redundancy reduction method, which was developed in our previous study [14].

## Results

### Performance measures

We performed a 10-fold cross validation to train and test the prediction models. For a more rigorous evaluation, we tested them on independent datasets that were not used in training them. The performance of the prediction models was evaluated with respect to six measures: sensitivity, specificity, accuracy, positive predictive value, negative predictive value, and Matthews correlation coefficient.

Sensitivity (SN) is the ratio of correctly predicted binding nucleotides to actual binding nucleotides (equation 5). Specificity (SP) is the ratio of correctly predicted non-binding nucleotides to actual non-binding nucleotides (equation 6). Accuracy (ACC) is the ratio of correctly predicted nucleotides to all nucleotides (equation 7). Positive predictive value (PPV) measures the ratio of correctly predicted binding nucleotides to all nucleotides that are predicted as binding (equation 8). Negative predictive value (NPV) measures the ratio of correctly predicted non-binding nucleotides to all nucleotides that are predicted as non-binding (equation 9). The Matthews correlation coefficient (MCC) is a strong indicator for multi-class problems and returns a score between -1 and 1 (equation 10).

Sensitivity, specificity and accuracy do not provide reliable performance indicators for imbalanced data. For example, consider a data set of 30 positives and 2,000 negatives which shows a sensitivity of 67%, a specificity of 91% and an accuracy of 91%. If it has nine times more false positives than true positives, the positive predictive value (PPV) can be as low as 10% despite the seemingly reasonable values of sensitivity, specificity and accuracy. Thus, we compute PPV and negative predictive value (NPV) in addition to sensitivity, specificity and accuracy.

In equations 6-10, the true positives (TP) are binding nucleotides that are correctly predicted as binding nucleotides, the true negatives (TN) are non-binding nucleotides that are predicted as non-binding nucleotides, the false positives (FP) are non-binding nucleotides that are incorrectly predicted as binding nucleotides, and the false negatives (FN) are binding nucleotides that are incorrectly predicted as non-binding nucleotides.

(5) $$\text{Sensitivity}=\frac{TP}{TP+FN}$$

(6) $$\text{Specificity}=\frac{TN}{TN+FP}$$

(7) $$\text{Accuracy}=\frac{TP+TN}{TP+TN+FP+FN}$$

(8) $$\text{PPV}=\frac{TP}{TP+FP}$$

(9) $$\text{NPV}=\frac{TN}{TN+FN}$$

(10) $$\text{MCC}=\frac{TP\times TN-FP\times FN}{\sqrt{\left(TP+FP\right)\left(TP+FN\right)\left(TN+FP\right)\left(TN+FN\right)}}$$

### Comparison of two SVM models

We performed a 10-fold cross validation for the two prediction models with various window sizes from 3 to 31 to examine the effect of window sizes on the prediction performance. As shown in Table 1, the prediction model that used both DNA and protein sequences (DPI2) showed better performance than the model that used only a DNA sequence (DPI1). DPI1 showed the best result with a window of size 23 (sensitivity of 74.01%, specificity of 72.92%, accuracy of 73.42%, PPV of 70.16%, NPV of 76.53% and MCC of 0.468). DPI2 showed the best result with a window of size 23 (sensitivity 80.8%, specificity 83.75%, accuracy 82.51%, PPV 78.27%, NPV 85.76% and MCC of 0.643).

**Table 1:** 10-fold cross validation with different window sizes (WS) from 21 to 31.

WS		DPI1						DPI2				
		
	SN (%)	SP (%)	ACC (%)	PPV (%)	NPV (%)	MCC	SN (%)	SP (%)	ACC (%)	PPV (%)	NPV (%)	MCC
21	73.78	72.77	73.24	70.01	76.31	0.464	80.71	83.49	82.32	77.99	85.65	0.639
23	**74.01**	**72.92**	**73.42**	**70.16**	**76.53**	**0.468**	**80.80**	**83.75**	**82.51**	**78.27**	**85.76**	**0.643**
25	73.78	73.12	73.42	70.22	76.45	0.468	80.85	83.48	82.38	77.99	85.76	0.640
27	73.56	73.14	73.33	70.15	76.32	0.466	80.78	83.70	82.47	78.19	85.75	0.642
29	73.37	73.05	73.20	70.02	76.18	0.463	80.85	83.65	82.47	78.15	85.79	0.642
31	72.79	73.46	73.15	70.16	75.99	0.462	80.86	83.62	82.46	78.12	85.79	0.642

SN: sensitivity, SP: specificity, ACC: accuracy, PPV: positive predictive value, NPV: negative predictive value, MCC: Matthews correlation coefficient.

In the test on an independent dataset that were not used in training, DPI2 showed a lower sensitivity than DPI1, but the other measures were higher than those of DPI1 (Table 2). In the test on the independent dataset, DPI1 showed the best result with a window of size 29 (73.39% sensitivity, specificity 64.81%, accuracy 68.86%, PPV 65.12%, NPV 73.11% and MCC 0.381). In the test on the independent dataset, DPI2 showed the best result with a window of size 31 (sensitivity of 67.61%, specificity of 74.27%, accuracy of 71.37%, PPV of 66.95%, NPV of 74.84% and MCC of 0.418).

**Table 2:** Testing on independent datasets with different window sizes (WS) from 21 to 31.

WS		DPI1						DPI2				
		
	SN (%)	SP (%)	ACC (%)	PPV (%)	NPV (%)	MCC	SN (%)	SP (%)	ACC (%)	PPV (%)	NPV (%)	MCC
21	73.39	64.29	68.59	64.79	73.00	0.377	66.41	73.89	70.63	66.22	74.05	0.403
23	73.02	64.09	68.31	64.55	72.63	0.372	66.55	74.00	70.75	66.36	74.16	0.405
25	72.15	64.48	68.11	64.53	72.11	0.366	66.76	74.16	70.94	66.57	74.32	0.409
27	73.17	64.61	68.65	64.93	72.89	0.378	67.39	74.10	71.18	66.74	74.67	0.415
29	**73.39**	**64.81**	**68.86**	**65.12**	**73.11**	**0.382**	67.39	74.27	71.28	66.88	74.71	0.416
31	73.17	64.94	68.82	65.14	72.99	0.381	**67.61**	**74.27**	**71.37**	**66.95**	**74.84**	**0.418**

SN: sensitivity, SP: specificity, ACC: accuracy, PPV: positive predictive value, NPV: negative predictive value, MCC: Matthews correlation coefficient.

**Figure 3:**
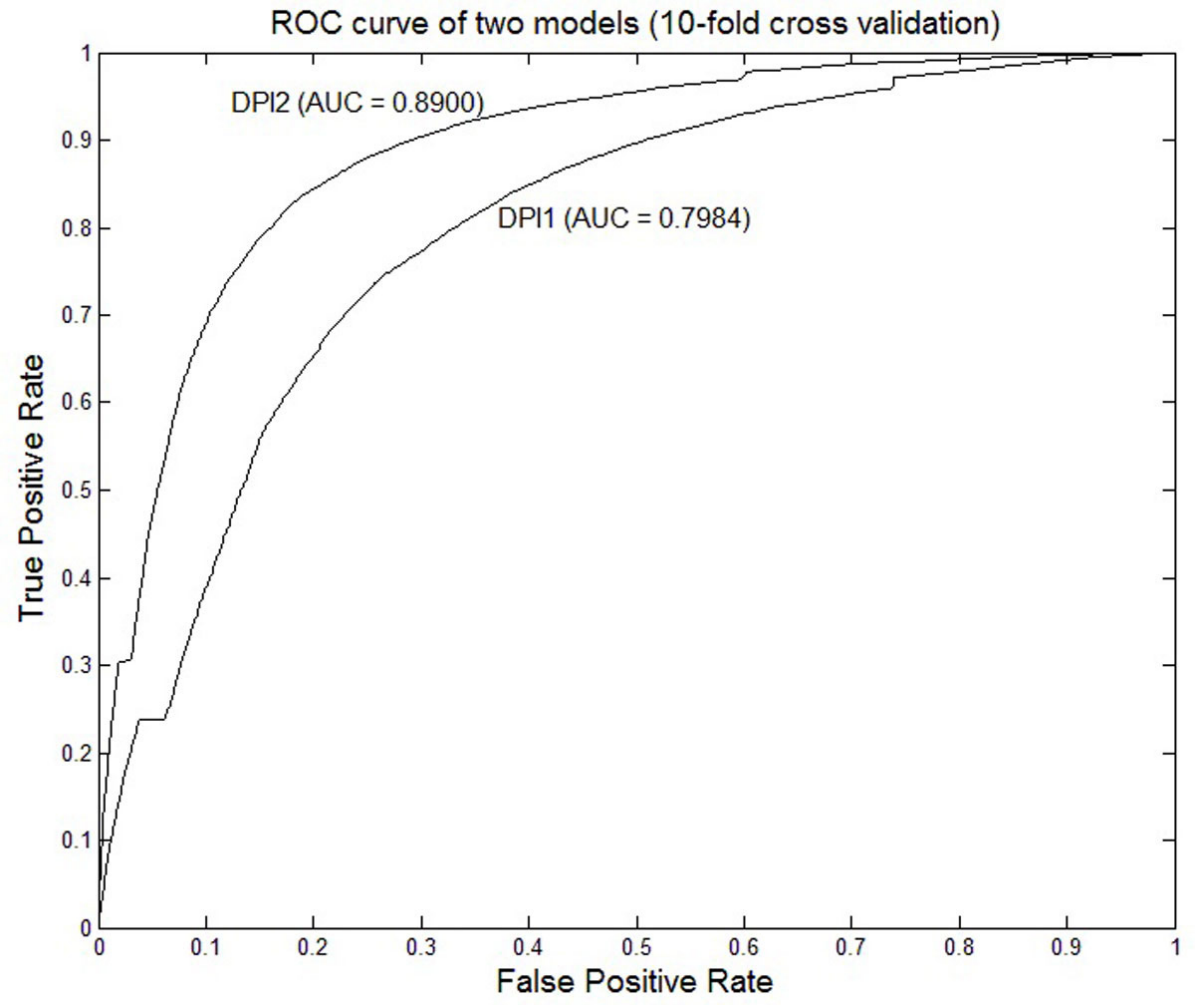
**ROC curves of the 10-fold cross validation of the two models. AUC: area under the ROC curve**. 10-fold cross validation results of the two models with a sliding window of size 23. AUC of DPI2 that uses both DNA and protein sequence data is higher than DPI1 that uses DNA sequence data alone.

**Figure 4:**
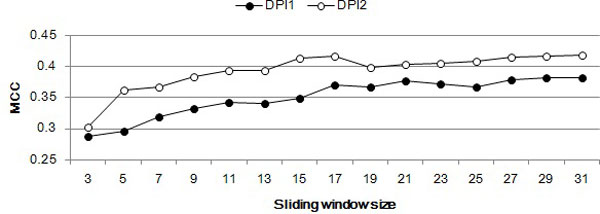
**Matthews correlation coefficient (MCC) of DPI1 and DPI2 with different window sizes**. MCC of DPI2 is higher than that of DPI1 in all window sizes. In both DPI1 and DPI2, MCC tends to increase as the window size increases, but it does not increase any more with a window of size 19 or larger.

Figure 3 shows ROC curves and their area under the curve (AUC) of the two models with a window of 23 nucleotides. AUC of DPI2 is larger than DPI1, indicating that DPI2, which used additional partner protein sequence data, was better than DPI1. As the window size increases, MCC tends to increase but does not increase any more with a window of size 19 or larger (Figure 4). This is because a larger window includes more null nucleotides at both ends of the window.

**Figure 5:**
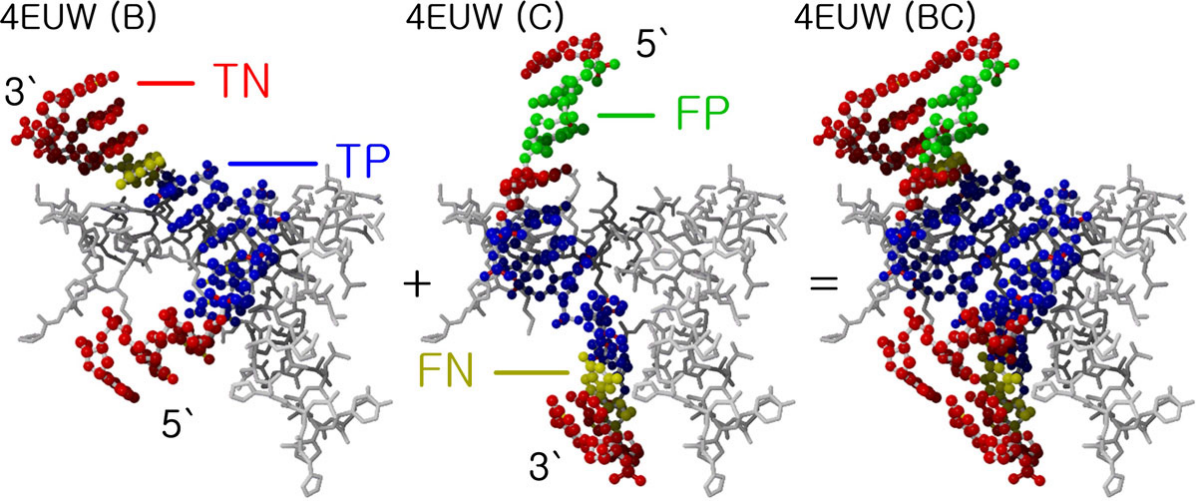
**Example of predicting protein binding sites in a double stranded DNA (PDB ID: **4EUW**)**. Blue balls, green balls, red balls and yellow balls represent true positives (TP), false positives (FP), true negatives (TN), and false negatives (FN), respectively. Protein is colored in gray. Chain B of 4EUW and chain C of 4EUW are complementary DNA sequences with the same partner protein, and their binding sites were predicted independently by the prediction model.

PNImodeler takes DNA sequence data as input. When PNImodeler is given a single DNA sequence instead of double-stranded DNA sequences as input, it can automatically generate its reverse complement sequence based on the base pairing rule and predict protein-binding nucleotides in both the input DNA and its reverse complement sequences. As an optional input data, PNImodeler takes a protein sequence that interacts with the DNA. Figure 5 shows an example of predicting protein binding sites in a double-stranded DNA.

Several programs have been developed to predict DNA-binding sites in proteins, but there are very few programs available that can predict protein-binding sites in DNA. PROMO [18] is one of the few programs that predict transcription factor (TF) binding sites in DNA sequences. For comparative purposes, we tested the two models of PNImodeler (DPI1 and DPI2) and PROMO on DNA sequences of recent **TF**-**DNA complexes **which were deposited into PDB after December 2013 (DNA chains D and E of 3WTV, DNA chains C and D of 4CHU, and DNA chains E and F of 4ON0). The model DPI2 of PNImodeler, which used both DNA and protein sequences, showed a sensitivity of 65.31%, a specificity of 75.33%, an accuracy of 71.43% and an MCC of 0.404 on average. The model DPI1 of PNImodeler, which used DNA sequences only, showed a sensitivity of 61.40%, a specificity of 77.47%, an accuracy of 70.31% and an MCC of 0.395 on average. With all listed transcription factors as candidate binding partners of the DNA sequences of the recent **TF**-**DNA complexes**, PROMO showed a sensitivity of 36.95%, a specificity of 71.42%, an accuracy of 57.08% and an MCC of 0.088 on average. These results demonstrate that PNImodeler is better than PROMO with or without the information on protein sequences.

## Conclusions

In general predicting protein binding sites in a double stranded molecule is more complex than predicting binding sites in single stranded molecules. We developed two SVM models to predict protein binding nucleotides in DNA. One model uses DNA sequence data alone and predicts all potential binding sites with unknown protein partners. The other model uses both DNA and protein sequences to predict protein binding nucleotides with the specific protein. In both 10-fold cross validation and independent testing, the second model that uses both DNA and protein sequences achieved better performance than the first model that uses DNA sequence data only.

We have implemented the SVM models as a web server called PNImodeler (Protein-Nucleic acid Interaction modeler), and it is available at http://bclab.inha.ac.kr/pnimodeler. This web server will be useful to find protein-binding sites in DNA with unknown structure. To the best of our knowledge, this is the first attempt to predict protein-binding DNA nucleotides with sequence data alone.

## Competing interests

The authors declare that they have no competing interests.

## Authors' contributions

JI performed experiments and prepared the first draft of the manuscript. BP analyzed protein-DNA interaction data and prepared data sets, and NT, WL, and DH gave comments on the manuscript. KH supervised the work and rewrote the manuscript.
